# Identification of overexpressed genes in *Sodalis glossinidius* inhabiting trypanosome-infected self-cured tsetse flies

**DOI:** 10.3389/fmicb.2014.00255

**Published:** 2014-05-27

**Authors:** Illiassou Hamidou Soumana, Bernadette Tchicaya, Béatrice Loriod, Pascal Rihet, Anne Geiger

**Affiliations:** ^1^IRD-CIRAD, UMR 177Montpellier, France; ^2^INSERM, UMR1090 TAGCMarseille, France; ^3^Biology Department, Aix-Marseille UniversityMarseille, France

**Keywords:** sleeping sickness, tsetse-symbiont-trypanosomes, tripartite interactions, control flies, self-cured tsetse flies

## Abstract

*Sodalis glossinidius*, one of the three tsetse fly maternally inherited symbionts, was previously shown to favor fly infection by trypanosomes, the parasites causing human sleeping sickness. Among a population of flies taking a trypanosome-infected blood meal, only a few individuals will acquire the parasite; the others will escape infection and be considered as refractory to trypanosome infection. The aim of the work was to investigate whether fly refractoriness could be associated with specific *Sodalis* gene expression. The transcriptome of *S. glossinidius* harbored by flies that were fed either with a non-infected blood meal (control) or with a trypanosome-infected meal but that did not develop infection were analyzed, using microarray technology, and compared. The analysis using the microarray procedure yielded 17 genes that were found to have a significant differential expression between the two groups. Interestingly, all these genes were overexpressed in self-cured (refractory) flies. Further analysis of functional annotation of these genes indicated that most associated biological process terms were related to metabolic and biosynthetic processes as well as to oxido-reduction mechanisms. These results evidence the occurrence of molecular crosstalk between the different partners, induced by the passage of the trypanosomes through the fly's gut even though the parasites were unable to establish in the gut and to develop a permanent infection.

## Introduction

Tsetse flies (*Glossina* spp.), the vectors of African trypanosomes causing sleeping sickness in humans (HAT, human African trypanosomiasis) and nagana (AAT, animal African trypanosomiasis) in animals, harbor symbiotic bacteria that regulate important aspects of their host's physiology. Two of these microbes, obligate *Wigglesworthia glossinidia* and commensal *Sodalis glossinidius*, are vertically transmitted (Cheng and Aksoy, 1999; Dale and Maudlin, 1999) to developing intrauterine progeny via maternal milk gland secretions (Attardo et al., 2008). Tsetse's third symbiont, *Wolbachia*, is transmitted via the germ-line cells (Cheng et al., 2000; Balmand et al., 2013). While the prevalence of *Wolbachia* infections is high in laboratory-reared fly colonies (Cheng et al., 2000), field population prevalence is much lower, and several tsetse fly species were never shown to harbor the symbiont (Doudoumis et al., 2012); in fact, we did not evidence the presence of *Wolbachia* in the population of *Glossina palpalis gambiensis* from which the individuals used in our experiments were selected (Geiger, personal communication). Nevertheless, the association between some tsetse fly species and the symbiont may have a long co-evolutionary history, since *Wolbachia* loci were found horizontally transferred into the host genome (Doudoumis et al., 2012). *S. glossinidius* is a secondary symbiont located intra-extracellularly in the fly's midgut, but it can be detected in other tissues (Cheng and Aksoy, 1999; Balmand et al., 2013). The association between *Sodalis* and tsetse fly was suggested to be recent (Chen et al., 1999). In the wild, the prevalence of fly infection by trypanosomes seldom exceed 10% of the population (Frézil and Cuisance, 1994; Maudlin and Welburn, 1994); similarly, when flies are fed with a trypanosome-infected blood meal in the insectary (Ravel et al., 2003), less than 50% of the flies will acquire the parasite and, as in field conditions, most will escape infection. This means that the normal status of the flies is to be refractory to trypanosome infection. As concerns *Sodalis*, this symbiont was believed to be involved in fly vector competence in enhancing the trypanosome susceptibility of its host, the tsetse fly (Welburn and Maudlin, 1999). In the wild, the presence of *Sodalis* has been demonstrated to favor fly infection by trypanosomes, assessing the suggested role of the symbiont in vector competence (Farikou et al., 2010). The suggested mechanism involved included the inhibition of the trypanocidal lectin, secreted by the fly during feeding, by N-acetyl glucosamine resulting from pupae chitin hydrolysis by chitinases secreted by the fly-hosted *S. glossinidius* (Maudlin and Ellis, 1985; Welburn et al., 1993; Welburn and Maudlin, 1999; Dale and Welburn, 2001). Finally, it was also shown that the effect of *S. glossinidius* could depend on its genotype (Geiger et al., 2007; Farikou et al., 2010).

However, an overview of the biological mechanisms by which, *in vivo*, the bacteria favors fly infection, and, conversely, the mechanisms by which the fly becomes refractory to trypanosome infection, is still lacking. In this context, the aim of the present work was to investigate whether fly refractoriness could be associated with specific *Sodalis* gene expression. Consequently, the transcriptomes of *S. glossinidius* harbored by flies that were fed either with a non-infected blood meal (control) or with a trypanosome-infected meal, but that did not develop infection, were analyzed using genome-wide *S. glossinidius* oligonucleotide microarrays and compared.

## Materials and methods

### Ethical statement

The experimental protocols involving animals were approved by the Ethics Committee and the Veterinary Department of the Centre International de Recherche Agronomique pour le Développement (CIRAD), Montpellier, France. The experiments were conducted according to internationally recognized guidelines.

### *Trypanosoma brucei gambiense* strain

The S7/2/2 *T. b. gambiense* strain used in this study was isolated in 2002 from HAT-affected patients living in the sleeping sickness focus of Bonon, Côte d'Ivoire (Ravel et al., 2006). The strain belongs to the homogenous *T. b. gambiense* group 1.

### Infection of *Glossina palpalis gambiensis*

The *G. p. gambiensis* flies used in this study originate from flies that were collected in the field in Burkina Faso. Pupae were collected from these flies. Following fly emergence, the population was maintained in a level-2 containment insectary at 23°C and 80% relative humidity (Geiger et al., 2005) without any selection. Individuals used in the present work were randomly chosen for infection experiments.

Experimental infections were conducted following the protocol reported by Ravel et al. (2006). *T. b. gambiense* stabilate was thawed at room temperature and 0.2 ml was injected intraperitoneally into balb/cj mice. The infection was monitored by examining tail blood using a phase-contrast microscope at a ×400 magnification. Teneral flies were then fed for the first time on infected mice displaying parasitemia levels between 15 and 25 × 10^7^ parasites/ml (determined using the matching method, Herbert and Lumsden, 1976). Ten days after infected blood-meal uptake, an anal drop was collected from each fly, and the fly infection status was determined by PCR examination using TBR specific primers (Moser et al., 1989) assessing the presence or absence of trypanosomes. Positive PCR results indicate trypanosome establishment in the fly midgut; negative PCR results indicate trypanosome self-cured flies. Less than 5% of the flies that were exposed to trypanosome were shown to be infected at day 10 post-infected blood-meal uptake. Only flies whose PCR result was negative were included in this study; they were designated as self-cleared or as refractory flies. Negative control samples consisted in teneral flies fed for the first time on non-infected mice. Finally, all the flies (fed on infected or non-infected mice) were later maintained by feeding on an uninfected rabbit, 3 days a week. Ten days after the first blood feeding (on either trypanosome infected or non-infected mice), the flies were dissected according to the method described by Penchenier and Itard (1981), and the samples, each of seven pooled midguts, respectively from control and refractory flies, were collected in 400 μl of RNA later (Ambion, France).

### RNA isolation

Total RNA was extracted from each sample using Trizol reagent (Invitrogen, France) according to the manufacturer's specifications. After extraction, RNA integrity was checked using agarose gel electrophoresis. The quality of RNA and the absence of any DNA contamination were checked on an Agilent RNA 6000 Bioanalyzer and quantified using the Agilent RNA 6000 Nano kit (Agilent Technologies, France).

### cDNA hybridization on microarray

RNA reverse transcription and fluorescent dye incorporation were carried out using the Promega ChipShot Direct Labeling and Clean-Up System (Promega, USA). For each sample, 5 μg of total RNA was reverse-transcribed and labeled with a single dye (Cy3) labeling procedure and used for microarray hybridization according to the manufacturer's indications (Promega). Each sample was run on custom-made 60-mers oligonucleotide microarrays specific for the *S. glossinidius* whole genome, and for the four plasmids (respectively, GenBank accession number, AP008232; NCBI RefSeq: NC_007183.1; NCBI RefSeq: NC_007184.1; NCBI RefSeq: NC_007186.1; NCBI RefSeq: NC_007187.1) with at least four oligonucleotide probes per gene (design is available at Gene Expression Omnibus under the accession number GPL17347). The Agilent design utilizes the uniqueness of probe sequences as one of the criteria for probe selection to avoid cross-hybridization with non-target genes. For each experimental condition four independent biological replicates were analyzed to ensure the high reproducibility and statistical significance of the expression data. The details of the expression data are available at Gene Expression Omnibus under accession number GSE48360.

### Microarray data analysis

The primary expression data were normalized through two successive steps using (a) both R software packages and lowess normalization to normalize the M-values for each array separately (within-array normalization) without prior background correction, and (b) quantile normalization to the *A*-values, making the density distributions similar across arrays to compare expression intensities between them (Bolstad et al., 2003). Normalized expression values were averaged through Cy3 signal intensities according to dye-swap replications to assign only one expression value to each biological replicate. Microarray data were scanned using an Agilent microarray scanner (Agilent Technologies), and the pictures were extracted with Agilent Feature Extraction software (version 10.5.1.1). Data were filtered for detectable expression level; only those showing a level of expression greater than the background noise in at least three of the four replicates were selected.

Unsupervised hierarchical clustering was used to investigate relationships between samples and between genes. It was applied to median-centered data, using the Cluster and TreeView programs (average linkage clustering using Pearson correlation as the metric distance). Statistical analysis was performed using the TMeV5 Multi Experiment Viewer, v4.5 software (http://www.tm4.org/mev.html) and two-class unpaired SAM (significant analysis of the microarray program) analysis method. One-way analysis of variance was applied to identify genes differentially expressed between infection self-cured and control flies. A 5% predicted false discovery rate was used as the threshold for differential expression (Reiner et al., 2003).

The identification of biological interpretation of differentially expressed genes was performed using DAVID software (Dennis et al., 2003). This program allows identification of the biological interpretation of genes in the basis of gene ontology (GO) terms. In addition, the Kyoto Encyclopaedia of Genes and Genomes (KEGG) pathways were used to assess the specific biological pathways that were overrepresented.

### Quantitative real-time PCR

The microarray results were controlled, using quantitative PCR (qPCR), on a subset of four genes (SG0845, SG0858, SG0895, and SG1978) that were shown to be differentially expressed in microarray experiments between the two groups of flies; these genes were among the highest overexpressed in refractory flies. Primers, specific to these genes (Table 1) were designed using Primer-Blast software (http://www.ncbi.nlm.nih.gov/tools/primer-blast/). cDNA was synthesized from 5 μg of original total RNA samples using random hexamers and Superscript II reverse-transcriptase (Invitrogen, France) according to the manufacturer's instructions. All qPCR reactions were performed in an Mx3005P QPCR System (Agilent Technologies) using the Brillant II Sybrgreen qPCR Kit (Agilent technologies) with 2 μl of cDNA of a known concentration in a 25-μl total volume. PCR efficiencies for each primer pair were calculated using tenfold dilutions of fly gut-extracted cDNA as previously described (Hamidou Soumana et al., 2013). PCR conditions were as follows: 94°C for 5 min (1×), followed by 94°C for 45 s, 60°C for 45 s, 72°C for 1 min (39×), and then 72°C for 10 min (1×). Melting curve analysis was performed to check the specificity of the PCR reaction and to verify the amplification efficiency. The housekeeping gene, *Glossina* tubulin (GenBank accession number HE861503), was used as the reference gene for the normalization calculation of relative expression quantification. Cycle thresholds (Ct) for each reaction were obtained using the MxPRO QPCR Software (Agilent Technologies). Relative quantification was calculated with the 2^−ΔΔ*C*(*t*)^ method as described by Livak and Schmittgen (2001). Relative quantification for given genes with respect to the calibrator was determined and compared with the normalized expression values resulting from microarray experiments.

**Table 1:** **Primers designed for microarray data confirmation by quantitative PCR (qPCR)**.

**Primer**	**Sequence (5′ → 3′)**	**Concentrations**	**Amplicon**
		**(nM)**	**size (bp)**
SG0845F	GCCAGCCTTATGTGGAAGGT	600	140
SG0845R	AGCCGGGGTGACTTTAGTTG	600	
SG0858F	GTTCATTCTCGGTCTGCCCA	600	136
SG0858R	GGCGGGTAAGCCGACATATT	600	
SG0895F	CGACCTGGTTATTAGCGGCA	300	121
SG0895R	CGCTACGTTATCCACCGACA	900	
SG1978F	ACCACTGCAACCGTTTTTCG	300	153
SG1978R	GCAGCTTATGACCGTCTCGT	150	
Gmm Tub F	CCATTCCCACGTCTTCACTT	600	149
Gmm Tub R	GACCATGACGTGGATCACAG	600	

Primers were designed with Primer-Blast software. The third and fourth columns represent forward and reverse primer concentration used in qPCR and PCR product size obtained, respectively.

## Results

The aim of the study was, using the microarray analysis procedure, to compare the transcriptome of *S. glossinidius* harbored by tsetse flies (*G. p. gambiensis*) that got an non-infected blood meal (control flies) with that of the symbiont harbored by flies that did not become infected despite they were fed with *T. b. gambiense* infected blood meal (refractory flies). The comparison is expected to allow to identify differential expressed genes, if any. The gene expression was analyzed 10 days after the flies had taken either their infective or a non-infective blood meal. Two-class SAM procedures were used to identify differentially expressed genes with a 5% false discovery rate.

### Identification of differentially expressed genes

Table 2 presents the 17 genes that exhibited significant differential expression between the two groups using the modified t-statistic SAM. Interestingly, all these genes were overexpressed in infection self-cured flies.

**Table 2:** **List of *S. glossinidius* significantly differentially expressed genes in microarray experiment using SAM procedure with a 5% false discovery rate**.

**Oligonucleotide**	**GenBank**	**Description**	**Fold**
**probe**	**accession**		**change**
	**number**		
SOD_P10175	SG2357	Phage capsid protein	1.5
SOD_P4185	SG0858__nagB	Glucosamine-6-phosphate deaminase	1.5
SOD_P4185	SG0858__nagB	Glucosamine-6-phosphate deaminase	1.4
SOD_P5905	SG1285	Hypothetical protein	1.5
SOD_P6235	SG1367	UTP-glucose-1-phosphate uridylyltransferase	1.7
SOD_P4337	SG0895	Galactokinase	1.5
SOD_P9701	SG2237	Serine/threonine protein kinase	1.3
SOD_P7410	SG1659	Hypothetical protein	1.3
SOD_P1886	SG0301__dipZ	Thiol:disulfide interchange protein	1.2
SOD_P7169	SG1597	NADH dehydrogenase I subunit F	1.4
SOD_P7309	SG1632	Lipoprotein precursor	1.3
SOD_P4186	SG0858__nagB	Glucosamine-6-phosphate deaminase	1.6
SOD_P4186	SG0858__nagB	Glucosamine-6-phosphate deaminase	1.7
SOD_P324	pSG1GP_81_triE	triE protein	1.6
SOD_P4134	SG0845	Phage tail sheath protein	1.8
SOD_P5250	SG1123	Bifunctional phosphoribosyl-AMP cyclohydrolase/phosphoribosyl-ATP pyrophosphatase	1.6
SOD_P1745	SG0267	ADP-ribose pyrophosphatase	1.4
SOD_P1747	SG0267	ADP-ribose pyrophosphatase	1.3
SOD_P4134	SG0845	Phage tail sheath protein	1.8
SOD_P8638	SG1972__recD	Exonuclease V subunit alpha	1.7
SOD_P4186	SG0858__nagB	Glucosamine-6-phosphate deaminase	1.5
SOD_P7310	SG1632	Lipoprotein precursor	1.3
SOD_P8638	SG1972__recD	Exonuclease V subunit alpha	1.4
SOD_P4690	SG0983	Hypothetical protein	1.7
SOD_P8662	SG1978	Prolipoprotein diacylglyceryl transferase	1.6
SOD_P4186	SG0858__nagB	Glucosamine-6-phosphate deaminase	1.5

The fold change represents the ratio of S. glossinidius gene expression level by comparing self-cured flies to control flies fed on a non-infected blood meal.

**Figure 1:**
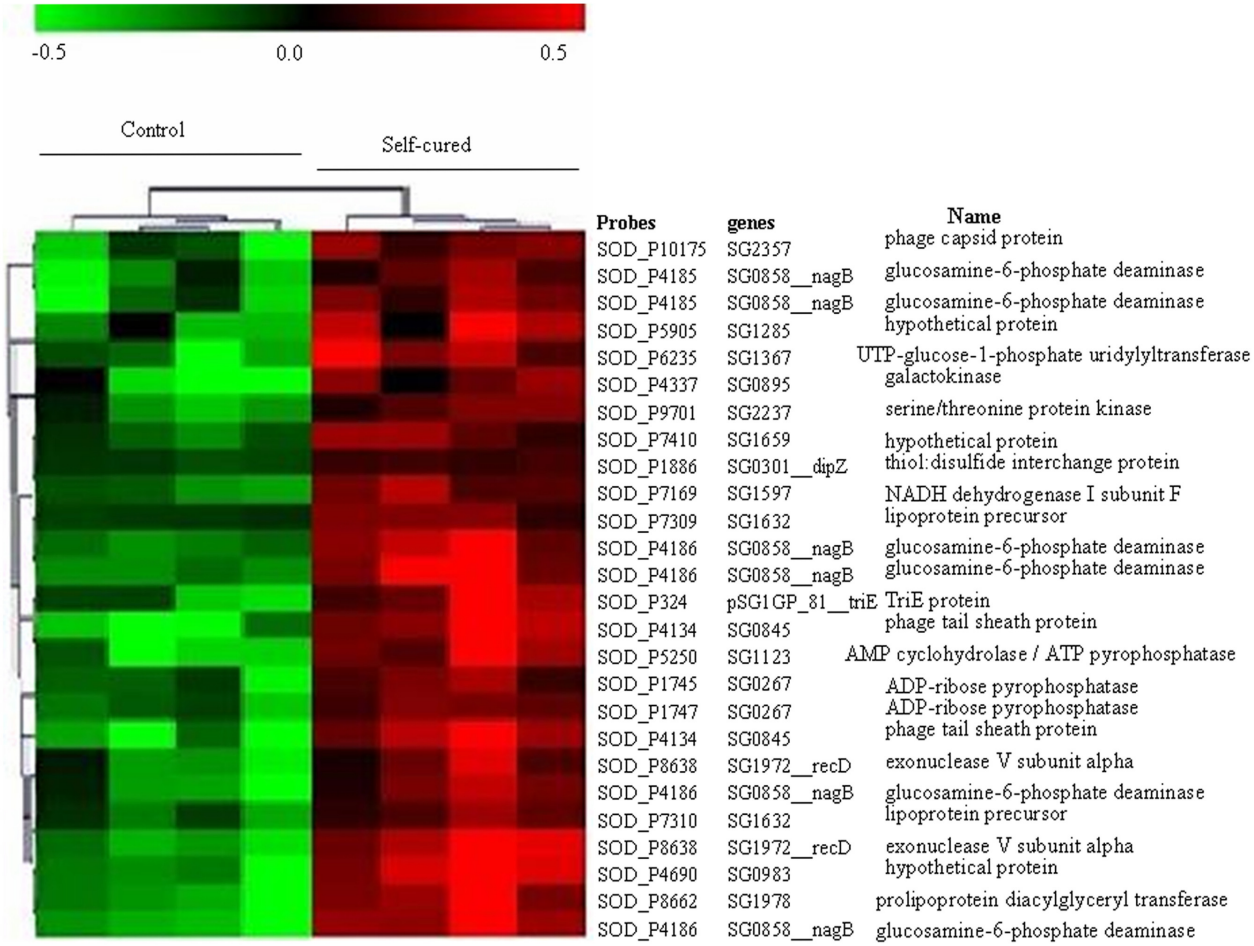
**Expression profile of *Sodalis glossinidius* genes whose transcript levels changed significantly between trypanosome self-cured flies and control flies fed on a non-infected blood meal**. This set of genes was extracted from the full data set (*n* = 2823) using a SAM procedure with a 5% false discovery rate. Each row represents a gene and each column represents a sample. Red and green indicate expression levels above and below the median, respectively. Dendrogram of genes, to the left of the matrix represents overall similarities in gene expression profiles.

We used an unsupervised hierarchical clustering method that grouped genes on the vertical axis and samples on the horizontal axis, on the basis of similarity in their expression profiles. The similarities are summarized in a dendrogram in which the pattern and length of the branches reflect the degree of relatedness of the samples (Figure 1).

According to microarray data, all significantly differentially expressed genes were overexpressed 1.2- to 1.8-fold in refractory flies with reference to the level of expression in control flies. The *S. glossinidius* gene (SG0858_nagB) corresponding to glucosamine-6-phosphate deaminase gene, is one of the most highly overexpressed in refractory flies (1.5- to 1.7-fold overexpression); this enzyme plays a crucial role in amino sugar and nucleotide sugar metabolism. We also detected increased expression levels of genes involved in purine metabolism such as ADP-ribose pyrophosphatase (SG0267; 1.4-fold increase), in D-galactose metabolism, represented by the gene encoding galactokinase (SG0895) and UTP-glucose-1-phosphate uridylyltransferase (SG1367), which were 1.5- and 1.7-fold over-represented in refractory flies. Oxidative respiration complex enzyme NADH dehydrogenase (SG1597) appears to be 1.4-fold overexpressed in refractory flies.

In resistant flies, we also found overexpressed genes involved in the exonucleolytic cleavage of DNA, synthesis of amino acids and lipoproteins, as well as in disulfide bond formation and assistance in the conformational maturation of secreted proteins containing disulfide bonds.

Finally, among the highest overexpressed genes, we identified genes coding for phage tail sheath protein (SG0845; 1.8-fold overexpression) and for phage capsid protein (SG2357; 1.5-fold overexpression).

### qPCR control of microarray data

**Figure 2:**
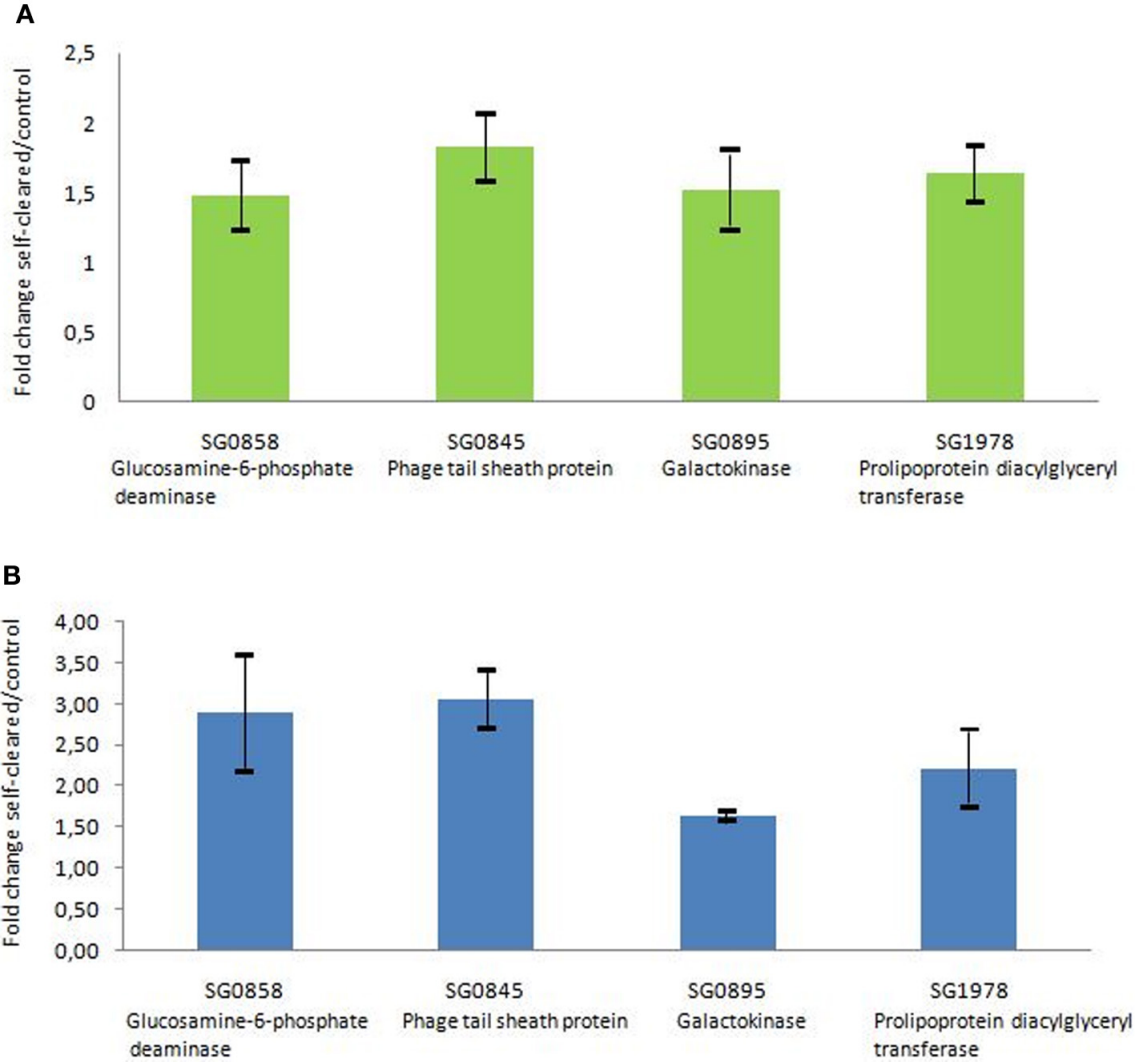
**Comparison of selected gene expression assessed by quantitative PCR and by microarray technologies. (A)** Gene expression was assessed by microarray technology. The n-fold change value was calculated on the basis of normalized data when comparing the level of gene expression from *S. glossinidius* derived from self-cured flies with those of control flies fed on a non-infected blood meal. Error bar represents the standard deviation (SD) between biological replicates. **(B)** Gene expression was assessed by quantitative PCR. Data were analyzed with the 2^−ΔΔ*C*(*t*)^ method with *Glossina* tubulin gene as a control gene. The n-fold change value represents the mean of the *Sodalis* gene expression level in self-cured flies compared with control. Error bar represents the SD between biological replicates.

The microarray expression data were validated by quantitative PCR analyses. Four genes showing different expression levels were selected from the microarray data. The results provided by the quantitative PCR analyses were similar to those provided by microarray data, showing consistent expression levels for the four genes (Figure 2).

### Gene functional annotation

Functional annotations of the differentially expressed *S. glossinidius* genes, with reference to the biological process GO terms and KEGG pathways, was investigated using DAVID software. The analysis showed an overrepresentation of GO terms related to metabolism and biosynthesis processes (Table 3). The modified Fisher exact test revealed a 17.6-fold enrichment (*P*-value = 0.055) for the GO term related to hexose metabolism (Table 3). Similarly, galactose metabolism KEGG pathways were found to be 28.5-fold-enriched (*P*-value = 0.088) (Table 4).

**Table 3:** **Biological process gene ontology (GO) terms associated with set of *S. glossinidius* significantly differentially expressed genes obtained with David software**.

**GO terms**		**Related gene**
		**GenBank accession**
		**number**
GO:0009249	Protein lipoylation	SG1978
GO:0018065	Protein-cofactor linkage	SG1978
GO:0042157	Lipoprotein metabolic process	SG1978
GO:0042158	Lipoprotein biosynthetic process	SG1978
GO:0005996	Monosaccharide metabolic process	SG0895
GO:0006012	Galactose metabolic process	SG0895
GO:0006793	Phosphorus metabolic process	SG0895
GO:0006796	Phosphate metabolic process	SG0895
GO:0016310	Phosphorylation	SG0895
GO:0019318	Hexose metabolic process	SG0895
GO:0046835	Carbohydrate phosphorylation	SG0895
GO:0000105	Histidine biosynthetic process	SG1123
GO:0006547	Histidine metabolic process	SG1123
GO:0008652	Cellular amino acid biosynthetic process	SG1123
GO:0009075	Histidine family amino acid metabolic process	SG1123
GO:0009076	Histidine family amino acid biosynthetic process	SG1123
GO:0009309	Amine biosynthetic process	SG1123
GO:0016053	Organic acid biosynthetic process	SG1123
GO:0018130	Heterocycle biosynthetic process	SG1123
GO:0044271	Nitrogen compound biosynthetic process	SG1123
GO:0046394	Carboxylic acid biosynthetic process	SG1123
GO:0005996	Monosaccharide metabolic process	SG1367
GO:0006006	Glucose metabolic process	SG1367
GO:0006011	UDP-glucose metabolic process	SG1367
GO:0009225	Nucleotide-sugar metabolic process	SG1367
GO:0019318	Hexose metabolic process^*^	SG1367
GO:0055114	Oxidation reduction	SG1597

* Enriched GO term with t-statistic modified test (P-value = 0.055)

**Table 4:** **Kyoto Encyclopaedia of Genes and Genomes (KEGG) pathways associated with set of *S. glossinidius* significantly differentially expressed genes obtained with David software**.

**KEGG**		**Related gene**
**pathways**		**GenBank accession**
		**number**
sgl00230	Purine metabolism	SG0267
sgl00052	Galactose metabolism^*^	SG0895
		SG1597
sgl00520	Amino sugar and nucleotide sugar metabolism	SG0895
sgl00340	Histidine metabolism	SG1123
sgl00040	Pentose and glucuronate interconversions	SG1367

* Enriched KEGG pathway with t-statistic modified test (P-value = 0.088)

## Discussion

After being ingested by a tsetse fly taking an infective blood meal, the trypanosome undergoes a complex cycle of differentiation and multiplication in the host midgut. Successful establishment of trypanosomes in the tsetse fly midgut depends on their ability to adapt, transform, grow, and survive rapidly in this new fly midgut environment (Simo et al., 2010). Several factors could influence parasite establishment, among which the tsetse midgut lectin (Welburn and Maudlin, 1999), reactive oxygen species (MacLeod et al., 2007), and antimicrobial peptide produced by the fly in response to trypanosome infection (Hao et al., 2001). Furthermore, *S. glossinidius*, was previously shown to favor tsetse fly infection by trypanosomes (Welburn and Maudlin, 1999). Despite the presence of the symbiont in all insectary tsetse flies, most of the flies are refractory to trypanosome infection (Geiger et al., 2005). In this context, we investigated the transcriptomic events that may occur in bacteria when they are harbored by refractory flies. Using microarray analysis we investigated *S. glossinidius* genes, the expression of which could discriminate *G. p. gambiensis* flies refractory to *T. b. gambiense* infection and control flies.

Most of the genes whose expression was modified, overexpressed in *Sodalis* from refractory versus *Sodalis* from control flies, are involved in lipoprotein metabolic and biosynthetic processes, as well as in amino sugar and nucleotide metabolism. Bacterial lipoproteins have been shown to play various roles, including nutrient uptake, transport (such as the ABC transport system), and extracytoplasmic folding of proteins (Lampen and Nielsen, 1984; Mathiopoulos et al., 1991; Alloing et al., 1994). The *Sodalis* prolipoprotein diacylglyceryl transferase gene (SG1978) that was found to be overexpressed in self-cured flies is the only one transferring the diacylglyceryl moiety to the thiol group of cysteine. The importance of this enzyme has been emphasized by the fact that post-translational modification is ubiquitous in the bacterial kingdom. The overexpression of enzymes involved in bacterial growth could be a necessary mechanism employed by the bacteria to fight the parasite.

The gene encoding the NADH dehydrogenase complex (SG1597) was found to be overexpressed in *Sodalis* from refractory flies. This enzyme is involved in the oxidative respiration process and allows bacteria to survive in a variety of hostile environments and to adapt quickly in a rapidly changing environment (Richardson, 2000). Furthermore, this enzyme is implicated in the synthesis of ATP, and thus energy metabolism in the prokaryotic cell (Lengeler et al., 2009). In mosquito cells, oxido-reduction mechanisms were used to protect against DENV viral infection (Patramool et al., 2011). Several other overexpressed genes in refractory flies were found to be involved in KEGG pathways and GO terms related to metabolism, such as galactose metabolism, purine metabolism, amino sugar and nucleotide metabolism, as well as hexose metabolism (Tables 3, 4). Thus, *S. glossinidius* might indeed benefit its tsetse host by nutrient supplementation via these compounds. However, why these pathways were overexpressed in refractory flies is unknown. In other studies, increased sugar metabolism enzyme activities due to viral infection have been reported (Klemperer, 1961; El-Bacha et al., 2004; Tchankouo-Nguetcheu et al., 2010). It has been suggested that the increased activity of glycolysis was due to the breakdown of the mitochondrial membrane, which decreased ATP production (Ritter et al., 2010). As a result, the glycolysis pathway was activated to compensate for the lack of energy via the oxidative pathway. However, recent studies have demonstrated alternative functions of sugar metabolism enzymes such as transcriptional regulation or as a regulator or indicator of apoptosis (Kim and Dang, 2005).

In addition, among the most highly overexpressed genes in refractory flies were those encoding glucosamine-6-phosphate deaminase (SG0858). This enzyme participates in amino sugar metabolism, particularly in the conversion of glucosamine into ammonium and fructose. *S. glossinidius* was suspected to favor trypanosome establishment in the insect midgut through a complex biochemical mechanism involving the production of N-acetyl glucosamine (Welburn and Maudlin, 1999). This sugar, resulting from hydrolysis of pupae chitin by a *S. glossinidius*-produced endochitinase was reported to inhibit a tsetse-midgut lectin lethal for the procyclic forms of the trypanosome (Dale and Welburn, 2001). So, while the presence of this sugar would favor the establishment of trypanosomes in the fly's midgut, its deamination by the glucosamine-6-phosphate deaminase may, in contrast, favor fly refractoriness. So in a next step, the decrease of N-acetyl glucosamine *in situ* will have to be studied.

Finally, increased transcription of genes coding phage proteins was recorded in refractory flies when compared to flies fed with a non-infected bloodmeal. These results are in line with those obtained previously when comparing refractory versus infected flies (Hamidou Soumana et al., 2014). Bacteriophage elements have also been identified in other symbiotic associations. So for the presence of bacteriophages APSE-1 and APSE-2 in the secondary endosymbiont of aphids ca. *Hamiltonella defensa* where they are associated with the protective activity of this secondary endosymbiont that kills parasitoid wasp larvae (Oliver et al., 2003; Moran et al., 2005; Degnan and Moran, 2008). Similarly, a bacteriophage, WO, was characterized in parasitic *Wolbachia*; the phage was suggested to be beneficial for the invertebrate host as it may be involved in the parasitic bacterial load regulation (Bordenstein and Wernegreen, 2004; Bordenstein et al., 2006). Finally a detailed characterization of mobile genetic elements and pseudogenes revealed the presence of different types of prophage elements that have proliferated across the genome of *S. glossinidius* (Belda et al., 2010). In addition, the presence of viral particles has been observed previously in *Sodalis glossinidius* cultures (Maudlin, personal communication).

Regarding our results, they highlight the probable role of a bacteriophage as a major actor in tsetse fly refractoriness. The activation of a prophage hosted by *S. glossinidius* could lead to the release of bacterial agonists that trigger the tsetse fly immune system preventing trypanosome development.

The overall results demonstrate the existence of a molecular dialog between the three partners—the fly, the symbiont, *Sodalis glossinidius*, and the trypanosome—even though the parasite could not establish in the fly's midgut. Some of the overexpressed genes belong to classical metabolic pathways; others, however, may be involved in fly refractoriness. The molecular signal that induces the overexpression of all these genes is unknown. Further investigations are needed to progress in the understanding of the complex tripartite interactions that control the fly vector competence and hence the spread of sleeping sickness.

### Conflict of interest statement

The authors declare that the research was conducted in the absence of any commercial or financial relationships that could be construed as a potential conflict of interest.
